# Apoptosis Repressor with a CARD Domain (ARC) Restrains Bax-Mediated Pathogenesis in Dystrophic Skeletal Muscle

**DOI:** 10.1371/journal.pone.0082053

**Published:** 2013-12-02

**Authors:** Jennifer Davis, Jennifer Q. Kwong, Richard N. Kitsis, Jeffery D. Molkentin

**Affiliations:** 1 Department of Pediatrics, Cincinnati Children’s Hospital Medical Center, University of Cincinnati, Cincinnati, Ohio, United States of America; 2 Department of Medicine and Wilf Family Cardiovascular Research Institute, Albert Einstein College of Medicine, Bronx, New York, United States of America; 3 Howard Hughes Medical Institute, Cincinnati, Ohio, United States of America; San Diego State University, United States of America

## Abstract

Myofiber wasting in muscular dystrophy has largely been ascribed to necrotic cell death, despite reports identifying apoptotic markers in dystrophic muscle. Here we set out to identify the contribution of canonical apoptotic pathways to skeletal muscle degeneration in muscular dystrophy by genetically deleting a known inhibitor of apoptosis, apoptosis repressor with a card domain (Arc), in dystrophic mouse models. *Nol3* (Arc protein) genetic deletion in the dystrophic *Sgcd* or *Lama2* null backgrounds showed exacerbated skeletal muscle pathology with decreased muscle performance compared with single null dystrophic littermate controls. The enhanced severity of the dystrophic phenotype associated with *Nol3* deletion was caspase independent but dependent on the mitochondria permeability transition pore (MPTP), as the inhibitor Debio-025 partially rescued skeletal muscle pathology in *Nol3* ^*-/-*^ *Sgcd* ^*-/-*^ double targeted mice. Mechanistically, *Nol3* ^*-/-*^ *Sgcd* ^*-/-*^ mice showed elevated total and mitochondrial Bax protein levels, as well as greater mitochondrial swelling, suggesting that Arc normally restrains the cell death effects of Bax in skeletal muscle. Indeed, knockdown of Arc in mouse embryonic fibroblasts caused an increased sensitivity to cell death that was fully blocked in *Bax Bak1* (genes encoding Bax and Bak) double null fibroblasts. Thus Arc deficiency in dystrophic muscle exacerbates disease pathogenesis due to a Bax-mediated sensitization of mitochondria-dependent death mechanisms.

## Introduction

 Muscular dystrophy is an inherited disorder characterized by skeletal muscle weakness and wasting that typically results in loss of ambulation with aging and premature death due to cardiac and respiratory dysfunction. The most common mutations fall within genes encoding structural or membrane proteins that are part of or influence the dystrophin-glycoprotein complex, which links the contractile apparatus within the cell to the extracellular matrix and in so doing, provides stability to the sarcolemma (plasma membrane of a skeletal muscle fiber). The loss of these structural components or their proper function renders the sarcolemma more susceptible to contraction induced permeation or rupture, which permits unrestrained Ca^2+^ entry [1-3]. The unregulated entry of Ca^2+^ is thought to be the primary initiator of skeletal muscle necrosis and subsequent inflammation and replacement fibrosis in muscular dystrophy [4].

 The mechanism whereby an unstable sarcolemma and unregulated Ca^2+^ influx causes skeletal myofiber death has been debated, and there is evidence that myofibers can die by apoptosis [5-8], necrosis [8-12], or both [8]. Several studies have identified TUNEL positive nuclei and caspase 3 activity in dystrophic skeletal muscle from both human and mouse [6,8,13,14] suggesting that muscle fibers can indeed die through apoptotic molecular effectors. While TUNEL positivity does not rule out necrotic cell death [15], caspase 3 activity is more highly indicative of apoptosis. By contrast the typical pathology characteristic of muscular dystrophy includes myofiber membrane rupture without containment of intracellular contents, fibrosis, and inflammation, all of which are features of necrotic cell death. Moreover the desensitization of mitochondrial permeability transition pore (MPTP) formation by both genetic deletion and pharmacologic inhibition of cyclophilin D (CypD) in several mouse models of muscular dystrophy showed reduced pathology and less muscle fiber death associated with this disease [11,16,17]. Collectively these studies demonstrate that a substantial proportion of muscle fiber wasting can be ascribed to a mitochondrial-dependent necrotic cell death process. However, Tidball et al. (1995) [8] suggested that early in the disease process *mdx* skeletal muscle fibers die by apoptosis, although this appeared to transition to a more necrotic cell death with age. It has also been proposed that secondary modifiers including reactive oxygen species, ischemia or environmental stimuli provide the signal that ultimately causes a muscle fiber to die by one pathway versus another [18]. To date the relative contribution of apoptotic versus necrotic cell death mechanisms to skeletal muscular dystrophy still remains inconclusive, although aspects of both molecular programs are clearly involved.

 To further investigate the molecular regulators of myofiber death in muscular dystrophy, we utilized a genetic approach by deleting the *Nol3* gene (encodes Arc, apoptosis repressor with a card domain) in several muscular dystrophy mouse models. Arc inhibits both the intrinsic and extrinsic apoptotic death pathways, where some of its targets are caspases 2 and 8 [19] as well as the proapoptotic Bcl-2 family member, Bax [20,21]. Arc is an extremely potent inhibitor of Bax as it directly binds this protein in the cytosol blocking its activation and translocation to the mitochondria [20,21]. This function of Arc is sufficient to restrain Bax activation and cell death during exposure to apoptotic stimuli *in vitro* [21]. However, Bax and Bak have more recently been suggested to also underlie necrotic cell death through effects on the mitochondria and MPTP [22,23]. Thus, Bax might be a convergence point at the level of the mitochondria that affects both apoptotic and necrotic pathways.

 In the heart, *Nol3* ^*-/-*^ mice showed increased signs of cell death, fibrotic remodeling and injury area following myocardial infarction (MI) or ischemia-reperfusion (IR) [24] providing further support for Arc’s protective role against cell death. Similarly *Nol3* ^*-/-*^ mice chronically exposed to a hypoxic environment exhibited a significant enhancement of arterial smooth muscle apoptosis [25]. While Arc can be found in several cell types [19], it is highly enriched in terminally differentiated cells that rarely undergo apoptosis, such as skeletal muscle. To date Arc deficiency has never been studied in muscular dystrophy; however, the forced expression of Arc protected both the heart and muscle derived H9c2 cell lines against death stimuli [20,26]. By contrast, skeletal muscle-specific transgenic overexpression of Arc in the *mdx* muscular dystrophy mouse model did not rescue its severe dystrophic phenotype [18], but this negative result might reflect the already saturating endogenous levels of Arc in skeletal muscle.

 Here we crossed *Nol3* ^*-/-*^ mice with either *Sgcd* ^*-/-*^ or *Lama2* ^*-/-*^ muscular dystrophy mouse models to create dystrophic skeletal muscles that are also devoid of Arc. In both models Arc deficiency heightened the severity of the dystrophic phenotypes as demonstrated by increased membrane permeability and areas of muscle wasting and fibrosis. Loss of Arc protein in *Sgcd* ^*-/-*^ mice revealed a more robust apoptotic biochemical signature in skeletal muscle compared with single *Sgcd* ^*-/-*^ controls, but treatment with the pan caspase inhibitor, zVAD-fmk, did not correct the enhanced muscle wasting in *Nol3 Sgcd* double null mice. By contrast, inhibition of MPTP-dependent cell death with Debio-025 significantly reduced the severity of the dystrophic phenotype characteristic of *Nol3 Sgcd* double null mice. Given Arc’s strong antagonistic relationship with Bax, we found that *Nol3 Sgcd* double null mice had increased total and mitochondrial levels of Bax in muscle, as well as greater mitochondrial swelling. shRNA-mediated knockdown of Arc also sensitized mouse embryonic fibroblasts (MEFs) to cell death stimuli, a result that was fully blocked in fibroblasts lacking Bax and Bak. Together these data implicate Bax-mediated mitochondrial mechanisms as responsible for dystrophic skeletal muscle pathology.

## Materials and Methods

### Animal Models & Treatments

 δ-sarcoglycan (*Sgcd* ^*-/-*^)[27], Arc (*Nol3* ^*-/-*^) [25,28], and laminin-2/merosin (*Lama2* ^*-/-*^) [29] gene-targeted mice were all previously described. In some studies zVAD-fmk (1.5 mg/kg in a 5% dimethylsulfoxide/ sterile saline vehicle; Abcam Biochemicals, Cambridge UK) was administered twice daily to *Nol3* ^*-/-*^ *Sgcd* ^*-/-*^ mice and wildtype (WT) littermates by intraperitoneal (i.p.) injection for 1 month beginning at the time of weaning. In other studies *Sgcd* ^*-/-*^ and *Nol3* ^*-/-*^ *Sgcd* ^*-/-*^ mice were treated with Debio-025 by gavage (80 mg/kg/day in a cremaphor-based vehicle; DebioPharm, Lausanne Switzerland) at weaning for 1 month. Littermates of both sexes were used for every experiment.

### Ethics Statement

 All animal experimentation was approved by the Office of Research Compliance and Regulatory Affairs and the Institutional Animal Care and Use Committee of the Cincinnati Children’s Hospital (Protocol Number: 2E11104). No human subjects were used

### Pathologic Indices

 For histological profiling muscles were fixed overnight in 10% formalin, paraffin embedded, and 7 µm sections were prepared for hematoxylin and eosin (H&E), Masson’s trichrome, and picrosirius red staining. Fibrosis was quantified by calculating the area of blue staining using Metamorph software (Molecular Devices LLC, Sunnyvale CA, USA). For collagen analysis, sections stained with picrosirius red were imaged and analyzed by polarized light. Serum creatine kinase (CK) levels were used as an index of muscle deterioration in the mouse models, which was performed by the clinical laboratory at the Cincinnati Children’s Hospital.

### TUNEL Assay

 Paraffin embedded muscle sections were deparaffinized, rehydrated, and permeabilized using 0.1% Triton X-100 and 0.1% sodium citrate buffer. The permeabilized sections were incubated for 1 hour in TUNEL (terminal deoxynucleotidyl transferase dUTP nick end-labeling) reaction mixture from an *In situ* cell death detection kit (Roche, Indianapolis IN, USA), washed, and incubated with TO-PRO3 nucleic acid stain (Invitrogen, Grand Island NY, USA). 

### Mitochondrial Swelling and Shrinking Assays

 Mitochondrial swelling and shrinking were assessed with light scattering measured at 540 nm over 10 minutes using 250 µg of mitochondria isolated from the hindlimbs of 4 week old mice. Hindlimb muscles were surgically removed and incubated for 30 minutes at 4°C in homogenization buffer (250 mM sucrose, 10 mM Tris, 1 mM EDTA pH 7.4) plus 1 mg/ml trypsin (Worthington Biochemical Corp, Lakewood NJ, USA). Muscles were minced and homogenized using glass-teflon homogenizers. The mitochondria were pelleted using differential centrifugation and the resulting pellet was resuspended in swelling buffer (120 mM KCl, 10 mM Tris, 5 mM KH_2_PO_4_, 7 mM pyruvate, and 1 mM malate). A 200 µM bolus of Ca^2+^ was used to initiate swelling and 5% polyethylene glycol (PEG) was used for shrinking. 

### Evans Blue Dye Assay

 Mice were injected with Evan’s blue dye (EBD) (10 mg/ml stock in sterile saline, 0.1 ml/10 g body weight) i.p. and 48 hours later they were euthanized and the skeletal muscles dissected and snap frozen in isopentane cooled OCT embedding media (Tissue-Tek, Sakura-Americas, Torrance CA, USA). Frozen OCT blocks were cryosectioned at 7 µm thickness and analyzed by fluorescence microscopy.

### Involuntary Running

 Six week-old mice were subjected to involuntary downhill treadmill running (Omni-Pacer LC4/M; Columbus Instruments International, Columbus OH, USA) for 30 minutes or until exhaustion. The treadmill was placed on a 15° decline to simulate downhill running. The mice were given a 10 minute acclimatization period at a speed of 6 m/min with the stimulation grid off. Following the acclimatization period the stimulation grid was turned on and the speed was progressively increased by 2 m/min every 3 minutes until a maximum speed of 18 m/min was attained. The criterion for exhaustion was when mice rested on the stimulation grid for longer than 5 consecutive seconds. Time to exhaustion and maximum speed were recorded for each subject.

### Western Blot

 Quadriceps muscles were isolated and snap frozen in liquid nitrogen for subsequent homogenization, or SV40 transformed MEFs were collected in lysis buffer (10 mM Tris-HCL, 150 mM NaCl, 4% glycerol, 0.5 mM NaMetabisulfite, 1% Triton X-100, 0.1% NaDeoxycholate, and 0.05% SDS). In some instances mitochondria were isolated from skeletal muscle by glass-teflon homogenization and centrifugation as previously described [30] and then prepared for Western blotting. Muscle, MEFs, and mitochondrial extracts were centrifuged at 13,000 *g* for 10 minutes and diluted in Laemmli buffer for SDS-PAGE separation. Western blotting with chemiluminescent detection was performed using the following primary antibodies and dilutions: Arc (1:500, rabbit polyclonal; Cayman Chemical, Ann Arbor MI, USA), Active Caspase 3 (1:500, rabbit polyclonal; BD Pharmingen, Franklin Lakes NJ, USA), Caspase 8 (1:500, mouse monoclonal; Cell Signaling, Danvers MA, USA), Bax (1:100, rabbit polyclonal; Santa Cruz Biotechnology, Santa Cruz CA, USA), Bak (1:800, rabbit polyclonal; BD-Biosciences, Franklin Lakes NJ, USA), Bcl-X_L_ (1:100, rabbit polyclonal; Santa Cruz), Actin (1:1000, mouse monoclonal; Sigma, St Louis MO, USA), Porin/VDAC (1:2000, mouse monoclonal; Mitoscience, Eugene OR, USA), GAPDH (1:5000, mouse monoclonal; Fitzgerald Industries, Acton MA, USA), α-actinin (1:500, mouse monoclonal; Sigma) and β-tubulin (1:100, mouse monoclonal; Santa Cruz Biotechnology). Goat-anti-mouse or goat-anti-rabbit conjugated alkaline-phosphatase (1:5000; Santa Cruz Biotechnology) secondary antibodies were used for detection. Western blots were quantified by densitometry using NIH Image J software and all values were normalized to a loading control. 

### Tissue Culture and Cell Death Assay

 WT and *Bax* ^*-/-*^ *Bak1* ^*-/-*^ SV40 transformed MEFs were maintained in Iscove’s modified Dulbecco’s Medium (IMDM) supplemented with 10% bovine growth serum, nonessential amino acids, and L-glutamine. For shRNA knockdown of the *Bax* (NM_007527) or *Nol3* (NM_030152) genes Mission shRNA lentiviruses (Sigma, Bax: TRCN0000009670-72 and Nol3: TRCN0000086915) were obtained and produced by the Heart Institute lentiviral vector screening core at Cincinnati Children’s Hospital Medical Center. Lentivirus was delivered in cultured media containing polybrene (8 μg/ml), and SV40 MEFs were incubated for 24 hours at which time the media was replaced with fresh. Knockdown was achieved at 4 days post gene transfer. For some experiments shBax knockdown MEFs and scrambled shRNA control MEFs were cultured with a proteasome inhibitor (50 nM Bortezomib, Selleck Chemicals, Houston TX, USA, or 50 μM MG-132, EMD Millipore, Billerica MA, USA) for 16 hours and collected for Western blot. For cell death assays MEFs (WT, *Bax* ^*-/-*^ *Bak1* ^*-/-*^, WT + shArc, *Bax* ^*-/-*^ *Bak1* ^*-/-*^ + shArc) were treated with 100 μM staurosporine (STS) for 12 hours. At which time MEFs were collected and incubated in annexin V-EGFP (AV) and propidium iodide (PI) (BioVision, Milpitas CA, USA) for 10 minutes. PI and AV positivity was quantified by flow cytometry (LSRII Flow Cytometer, BD Biosciences).

### Statistical Tests

 Statistical significance was determined by ANOVA and Newman-Keuls pairwise comparisons for multivariate experiments and t-test for experiments with 2 groups.

## Results

### Arc deficiency worsens dystrophic skeletal muscle pathology

**Figure 1:**
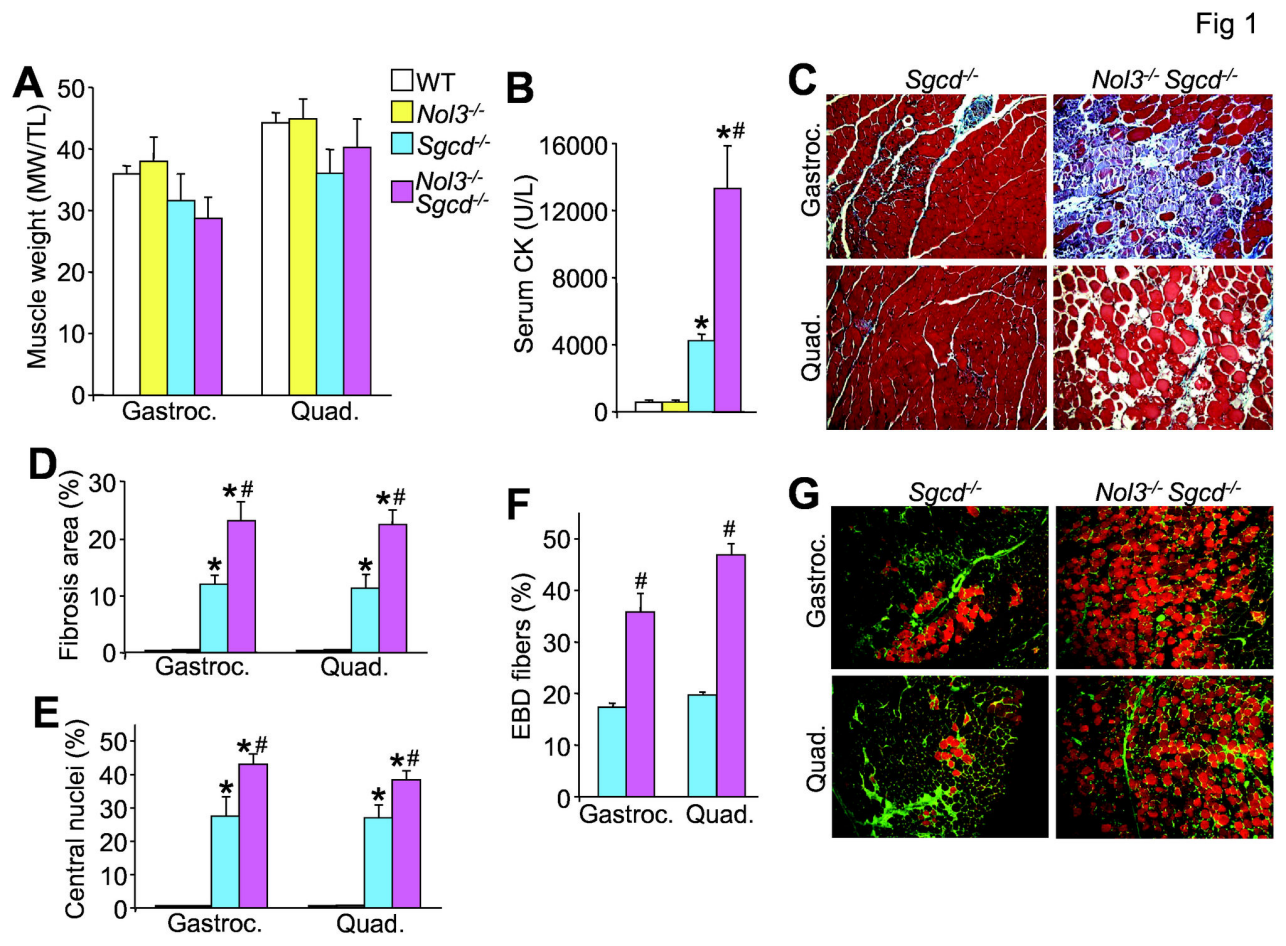
Nol3^-/-^Sgcd^-/-^ mice have enhanced skeletal muscle pathology relative to Sgcd^-/-^ mice at 4 weeks of age. A, Muscle weights normalized to tibial length of gastrocnemius and quadriceps, and B, serum creatine kinase (CK) levels measured from wildtype (WT), Arc null (Nol3^-/-^), Sgcd^-/-^, or double null (Nol3^-/-^Sgcd^-/-^) mice. *P<0.05 vs WT; #P<0.05 vs Sgcd^-/-^; N=8-15 per group. C, Images taken at 200x of Masson’s trichrome-stained sections of gastrocnemius and quadriceps from Sgcd^-/-^ and Nol3^-/-^Sgcd^-/-^ mice. The blue areas are collagen and fibrotic. D, Quantitation of the muscle fibrotic area by Metamorph software. *P<0.05 vs WT; #P<0.05 vs Sgcd^-/-^; N=5 per group. E, Quantitation of the number of fibers with central nucleation relative to total fiber number in all experimental groups for the muscles shown. *P<0.05 vs WT; #P<0.05 vs Sgcd^-/-^; N=5 per group with the identical 4 quadrants of the muscle counted per group. F, Quantification of Evan’s blue dye (EBD) positive myofibers in gastrocnemius and quadriceps from Sgcd^-/-^ and Nol3^-/-^Sgcd^-/-^ mice. #P<0.05 vs Sgcd^-/-^; N=4 per group with the identical 4 quadrants of the muscle counted per group. G, Histological images at 200x of EBD in orange and wheat germ agglutinin conjugated to FITC (green), the latter of which shows the muscle membrane.

 Mice lacking δ-sarcoglycan (*Sgcd* ^*-/-*^) were used as a robust model of muscular dystrophy [27,31]. To further examine the underlying molecular effectors of cell death in dystrophic myofibers here we crossed *Sgcd* ^*-/-*^ mice with mice lacking the gene encoding Arc (*Nol3* ^*-/-*^) [25,28] to obtain double nulls (Nol3^-/-^ *Sgcd* ^*-/-*^). Quadriceps muscle lysates were analyzed by Western blotting to verify the loss of Arc in Nol3^-/-^ *Sgcd* ^*-/-*^ mice versus controls. Arc protein expression was absent in muscle from double nulls, but Arc was slightly elevated in muscle from *Sgcd* ^*-/-*^ alone when compared to WT (Figure S1A). While there were no differences in the weights of gastrocnemius and quadriceps from any of the groups at 4 weeks of age (Figure 1A), serum CK levels in Nol3^-/-^ *Sgcd* ^*-/-*^ mice were approximately triple the significantly elevated values measured in *Sgcd* ^*-/-*^ mice (Figure 1B) indicating greater muscle damage in double null mice. The loss of Arc protein in muscle from *Sgcd* ^*-/-*^ mice also noticeably enhanced myofiber death in both the gastrocnemius and quadriceps (Figure 1C), producing a doubling in fibrotic area (Figure 1C and D and Figure S1B). The number of fibers with centrally located nuclei was also increased in muscle from Nol3^-/-^ *Sgcd* ^*-/-*^ mice, suggesting that the loss of Arc in muscular dystrophy results in more myofibers undergoing degeneration (Figure 1E). Indeed, EBD uptake, which marks both necrotic fibers and fibers with ruptured membranes, was increased to a significantly greater extent in Nol3^-/-^ *Sgcd* ^*-/-*^ compared with *Sgcd* ^*-/-*^ mice (Figure 1F and G). As an important control, *Nol3* ^*-/-*^ mice alone showed no muscle pathology or increased EBD uptake (Figure 1A, B, D and E and data not shown). While the analysis presented thus far was performed at 4 weeks of age, a nearly identical set of data were obtained at 6 weeks of age with significantly greater disease in Nol3^-/-^ *Sgcd* ^*-/-*^ mice compared with *Sgcd* ^*-/-*^ mice (Figure S2A-F). Loss of Arc even generated pseudohypertrophy in the gastrocnemius and quadriceps of *Sgcd* ^*-/-*^ mice at this slightly later age, indicating more substantial disease, and double nulls had significantly worse function when subjected to treadmill running (Figure S2A and F). Thus, loss of Arc dramatically enhances myofiber death and subsequent dystrophic disease in *Sgcd* ^*-/-*^ mice.

### *Nol3* ^*-/-*^ *Sgcd* ^*-/-*^ mice have increased molecular markers of apoptotic cell death

**Figure 2:**
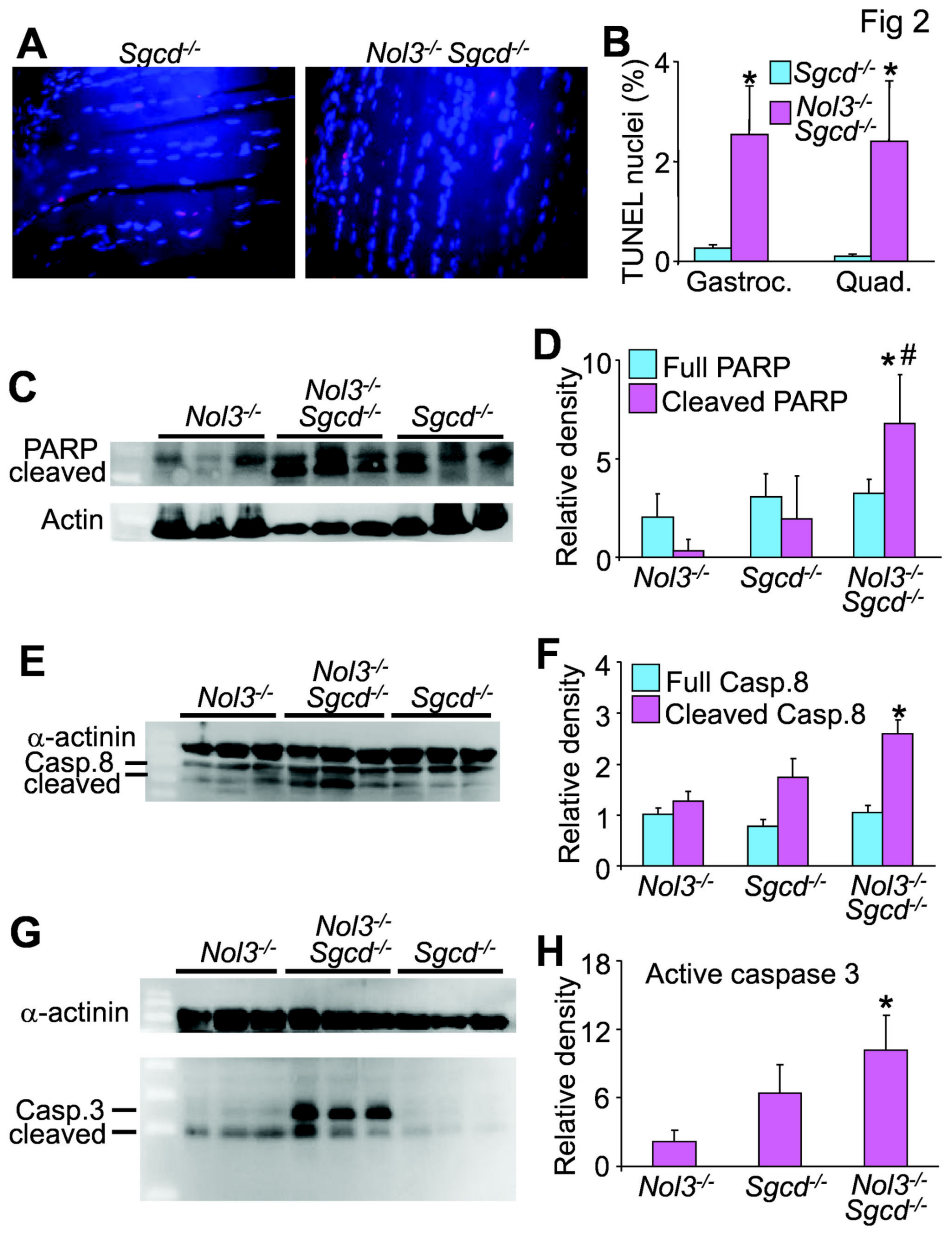
Arc deficiency in Sgcd^-/-^Nol3^-/-^ mice alters some markers of muscle apoptosis. A, Images of TUNEL staining (red) in longitudinal sections from quadriceps of 4 week-old mice. DAPI shows nuclei in blue. B, Quantification of TUNEL positive nuclei in gastrocnemius and quadriceps, which was determined by taking the relative proportion of TUNEL positive to normal nuclei only in a skeletal muscle fibers. *P<0.05 vs Sgcd^-/-^; N=6 per group with the identical 4 quadrants of the muscle counted per group. C, Western blot and D, quantification for the 85 kDa fragment of cleaved PARP from quadriceps lysates of Nol3^-/-^, Sgcd^-/-^ and Nol3^-/-^Sgcd^-/-^ mice (Actin serves as a loading control). *P<0.05 vs Nol3^-/-^; #P<0.05 vs Sgcd^-/-^; N=3 per group. E, Western blot and F, quantification of full-length (57 kDa) and cleaved caspase 8 (43 kDa) from quadriceps lysates of Nol3^-/-^, Sgcd^-/-^, and Nol3^-/-^Sgcd^-/-^ mice (α-actinin serves as a loading control). *P<0.05 vs Nol3^-/-^; N=3 per group. G, Western blot and H, quantification of cleaved caspase 3 (17 and 12 kDa) from quadriceps lysates of Nol3^-/-^, Sgcd^-/-^, and Nol3^-/-^Sgcd^-/-^ mice (α-actinin serves as a loading control). *P<0.05 vs Nol3^-/-^; N=3 per group.

 Because Arc has been identified as an inhibitor of apoptotic death, quadriceps and gastrocnemius muscles from 4 week-old Nol3^-/-^Sgcd^-/-^ mice were examined by TUNEL staining to identify if Arc deficiency enhances this molecular index of presumed apoptotic cell death in muscular dystrophy. Muscles were sectioned longitudinally in order to differentiate TUNEL positive myonuclei from non-muscle nuclei (Figure 2A). Both the quadriceps and gastrocnemius muscles of Nol3^-/-^ *Sgcd* ^*-/-*^ mice had significantly more TUNEL positive nuclei per fiber than *Sgcd* ^*-/-*^ mice (Figure 2B). The caveat with these data are that necrotic cell death can also lead to TUNEL positivity, but overall it can indicate total cell death burden. We also assessed both the intrinsic and extrinsic apoptosis pathways by Western blot analysis of the main active proteases. Cleaved poly(ADP-ribose) polymerase (PARP) was elevated in *Nol3* ^*-/-*^ *Sgcd* ^*-/-*^ muscle when compared to both *Nol3* ^*-/-*^ and *Sgcd* ^*-/-*^ (Figure 2C and D). Additionally, a significant increase in both cleaved caspase 8 (Figure 2E and F) and caspase 3 (Figure 2G and H) was detected in *Nol3* ^*-/-*^ *Sgcd* ^*-/-*^ muscle when compared to *Nol3* ^*-/-*^ but not *Sgcd* ^*-/-*^ muscle. Collectively, these data suggest that both the intrinsic and extrinsic arms of the apoptosis pathways are more active in *Nol3* ^*-/-*^ *Sgcd* ^*-/-*^ muscle. However, there was no detectable difference in cleaved caspase 8 and 3 between *Nol3* ^*-/-*^ *Sgcd* ^*-/-*^ and *Sgcd* ^*-/-*^ muscle suggesting that caspase activity is not a primary causal factor for the increased muscle pathology in *Nol3* ^*-/-*^ *Sgcd* ^*-/-*^ mice. Thus, while greater cell death is clearly occurring in skeletal muscle from Nol3^-/-^Sgcd^-/-^ mice compared with *Sgcd* ^*-/-*^ mice, the death is unlikely to be a result of bona fide apoptosis (see below for more definitive experiments to address this point). 

### Loss of Arc accelerates the onset of pathology in a mouse model of congenital muscular dystrophy (Lama2^-/-^)

**Figure 3:**
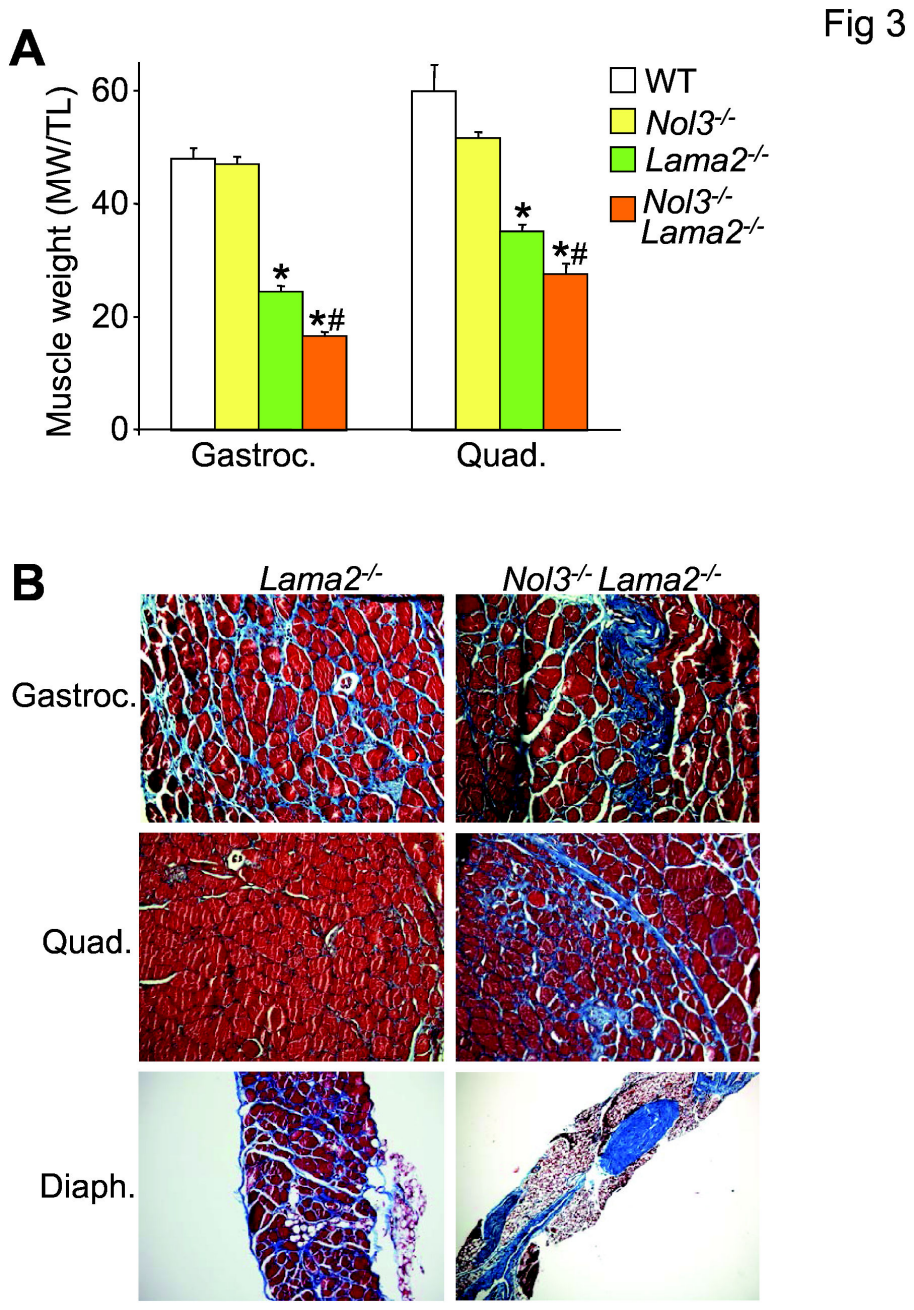
Nol3^-/-^Lama2^-/-^ mice have smaller skeletal muscles and more severe pathology. A, Muscle weights normalized to tibial length of gastrocnemius and quadriceps measured from WT, Nol3^-/-^, Lama2^-/-^, and Nol3^-/-^Lama2^-/-^ mice. *P<0.05 vs WT; #P<0.05 vs Lama2^-/-^; N=9 per group. B, Histological images taken at 200x of Masson’s trichrome stained sections of gastrocnemius, quadriceps and diaphragm from Lama2^-/-^ and Nol3^-/-^Lama2^-/-^ mice.

 We also extended our analysis to a mouse model of congenital muscular dystrophy that is known to have very severe disease as well as a link to Bcl-2 family member-mediated cell death [32]. Here loss of Arc protein in the dystrophic *Lama2* ^*-/-*^ background (Nol3^-/-^Lama2^-/-^) caused a significantly greater decrease in gastrocnemius and quadriceps muscle weight when compared to the already small muscles in *Lama2* ^*-/-*^ mice at 4 weeks of age, due to high levels of necrosis (Figure 3A). Nol3^-/-^Lama2^-/-^ mice also had increased fibrosis throughout these muscles (Figure 3B). The diaphragm was the most severely affected as there was a dramatic loss of myofibers in the Nol3^-/-^ Lama2^-/-^ mice (Figure 3B). Collectively, these data suggest that the loss of Arc further exacerbates myofiber death in the *Lama2* null mice.

### Caspase inhibition does not improve the dystrophic pathology in *Nol3* ^*-/-*^ *Sgcd* ^*-/-*^ mice

**Figure 4:**
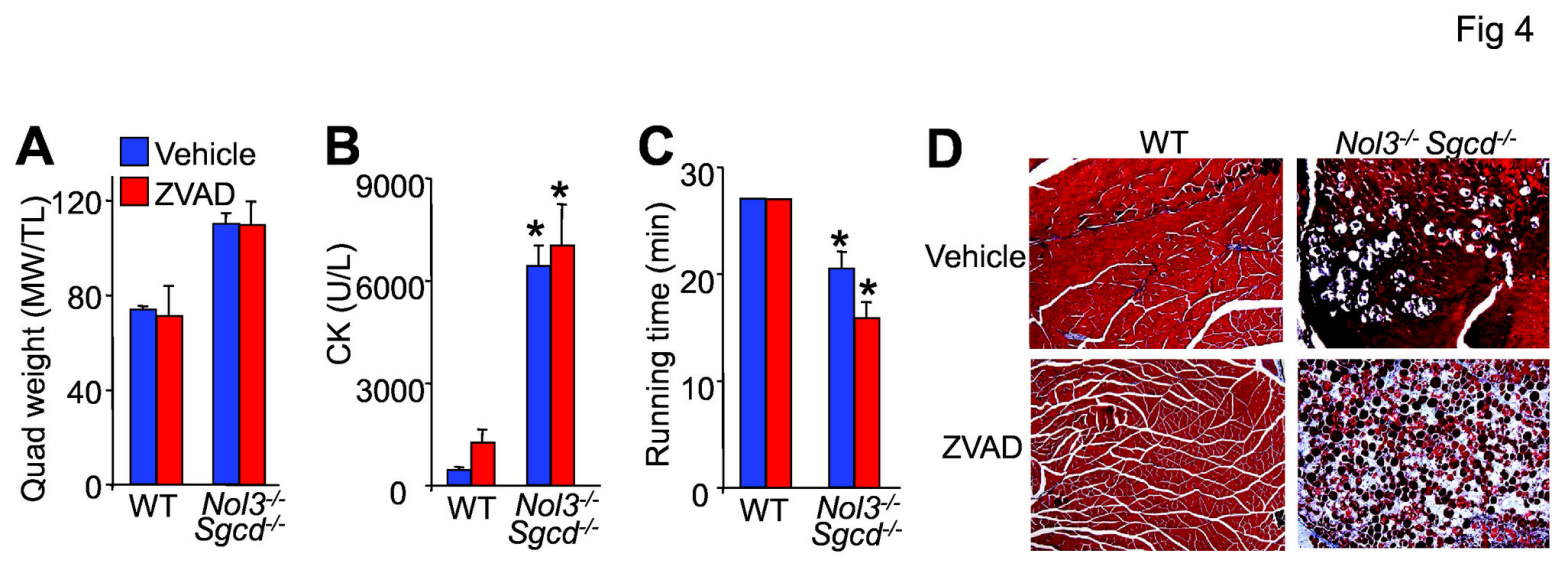
ZVAD-fmk treatment does not correct muscular dystrophy in Nol3^-/-^Sgcd^-/-^ mice. A, Muscle weights normalized to tibial length of quadriceps of mice treated with or without ZVAD-fmk for 4 weeks. B, CK levels, and C, quantification of the time to exhaustion as assessed by involuntary treadmill running measured from vehicle or ZVAD-fmk treated WT and Nol3^-/-^ Sgcd^-/-^ mice. *P<0.05 vs WT; N=4 per group. D, Histologic images taken at 100x of Masson’s trichrome stained sections of quadriceps from vehicle or ZVAD-fmk treated WT and Nol3^-/-^Sgcd^-/-^ mice.

 Arc has been previously shown to inhibit both the extrinsic and intrinsic apoptotic pathways through caspase and Bax-dependent mechanisms, respectively [19-21,26]. WT and Nol3^-/-^Sgcd^-/-^ mice were treated at weaning with the pan caspase inhibitor zVAD-fmk (1.5 mg/kg) twice daily for 4 weeks. Caspase inhibition did not correct the muscle pseudohypertrophy (Figure 4A) or the significantly elevated serum CK levels in Nol3^-/-^Sgcd^-/-^ mice (Figure 4B). Nol3^-/-^Sgcd^-/-^ mice were also functionally assessed by forced-treadmill running, which showed no correction in their poor running performances with zVAD-fmk treatment (Figure 4C). Finally, zVAD-fmk did not improve the severe fibrosis and myofiber fiber drop-out observed in skeletal muscle from Nol3^-/-^Sgcd^-/-^ mice (Figure 4D). These results further suggest that caspase activity as linked to the apoptotic pathway is not a significant contributor to the pathologic mechanisms underlying muscular dystrophy in this mouse model of disease. 

### Inhibition of MPTP-dependent cell death by Debio-025 reduces muscle pathology in *Nol3* ^*-/-*^ *Sgcd* ^*-/-*^ mice

**Figure 5:**
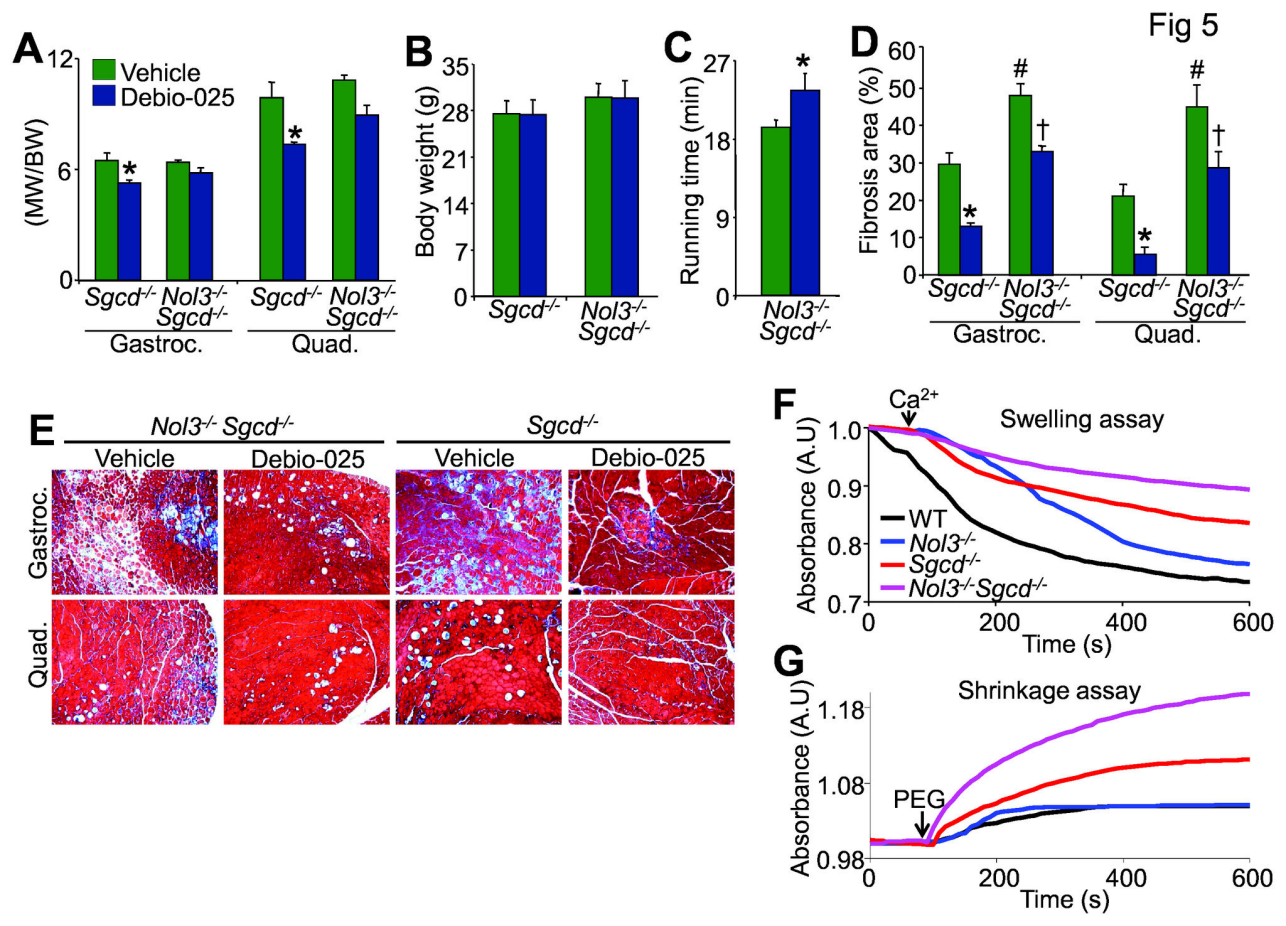
Treatment with the MPTP inhibitor Debio-025 reduces skeletal muscle pathology in Nol3^-/-^Sgcd^-/-^ mice. A, Muscle weights normalized to body weight of gastrocnemius and quadriceps, and B, body weight measurements of Sgcd^-/-^ and Nol3^-/-^Sgcd^-/-^ mice treated with vehicle or Debio-025 for 4 weeks. C, Quantification of the time to exhaustion as assessed by involuntary treadmill running measured from vehicle or Debio-025 treated Nol3^-/-^ Sgcd^-/-^ mice. D, Quantitation of fibrosis and associated E, histological images taken at 100x of Masson’s trichrome stained sections in gastrocnemius and quadriceps muscles from vehicle or Debio-025 treated Sgcd^-/-^ and Nol3^-/-^Sgcd^-/-^ mice. *P<0.05 vs vehicle; #P<0.05 vs Sgcd^-/-^ + vehicle; †P<0.05 vs Nol3^-/-^Sgcd^-/-^ + vehicle; N=5-7 per group. Representative absorbance (540 nm) readings from skeletal muscle derived mitochondria demonstrating the amount of mitochondrial F, swelling in response to Ca^2+^ and G, shrinkage in response to PEG from the indicated genotypes of mice.

 Debio-025, a cyclophilin inhibitor, has been shown to improve muscle pathology associated with several mouse models of muscular dystrophy, including *Sgcd* ^*-/-*^ mice, through its action of preventing MPTP-induced myofiber necrosis [11,17]. Here, Debio-025 treatment for 1 month significantly reduced muscle weights due to pseudohypertrophy in *Sgcd* ^*-/-*^ mice (Figure 5A), with a trend towards a decrease in Nol3^-/-^Sgcd^-/-^ mice, without affecting body weight (Figure 5A and B). While these data are suggestive, Debio-025 did significantly improve the poor running performance of Nol3^-/-^Sgcd^-/-^ mice (Figure 5C) and significantly reduce muscular fibrosis in both single and double null mice (Figure 5D and E). To ascertain if the Nol3^-/-^Sgcd^-/-^ muscle pathology is due to mitochondrial dysfunction we analyzed mitochondrial swelling and shrinking (Figure 5F and 5G). Mitochondria isolated from 4 week-old *Sgcd* ^*-/-*^ muscle were refractory to Ca^2+^ (200 μM) induced swelling relative to WT and *Nol3* ^*-/-*^, while Nol3^-/-^Sgcd^-/-^ mitochondria had an even greater impairment (Figure 5F). We also used polyethylene glycol (PEG, 5%) to induce hyperosmotic shock and found that Nol3^-/-^Sgcd^-/-^ mitochondria shrink more than those from *Sgcd* ^*-/-*^ muscle (Figure 5G). Hyperosmotic shock also caused *Sgcd* ^*-/-*^ mitochondria to shrink more than WT and *Nol3* ^*-/-*^ mitochondria (Figure 5G) similar to previously published reports [11,30]. Taken together these data demonstrate that Nol3^-/-^Sgcd^-/-^ mitochondria are indeed more swollen and hence more dysfunctional than *Sgcd* ^*-/-*^ mitochondria, which are preswollen at baseline even with functional *Nol3*. These data suggest that MPTP-dependent myofiber necrosis significantly contributes to the severe muscle pathology observed in Nol3^-/-^Sgcd^-/-^ mice.

### Arc deficiency results in a Bax-mediated sensitization to cell death stimuli

**Figure 6:**
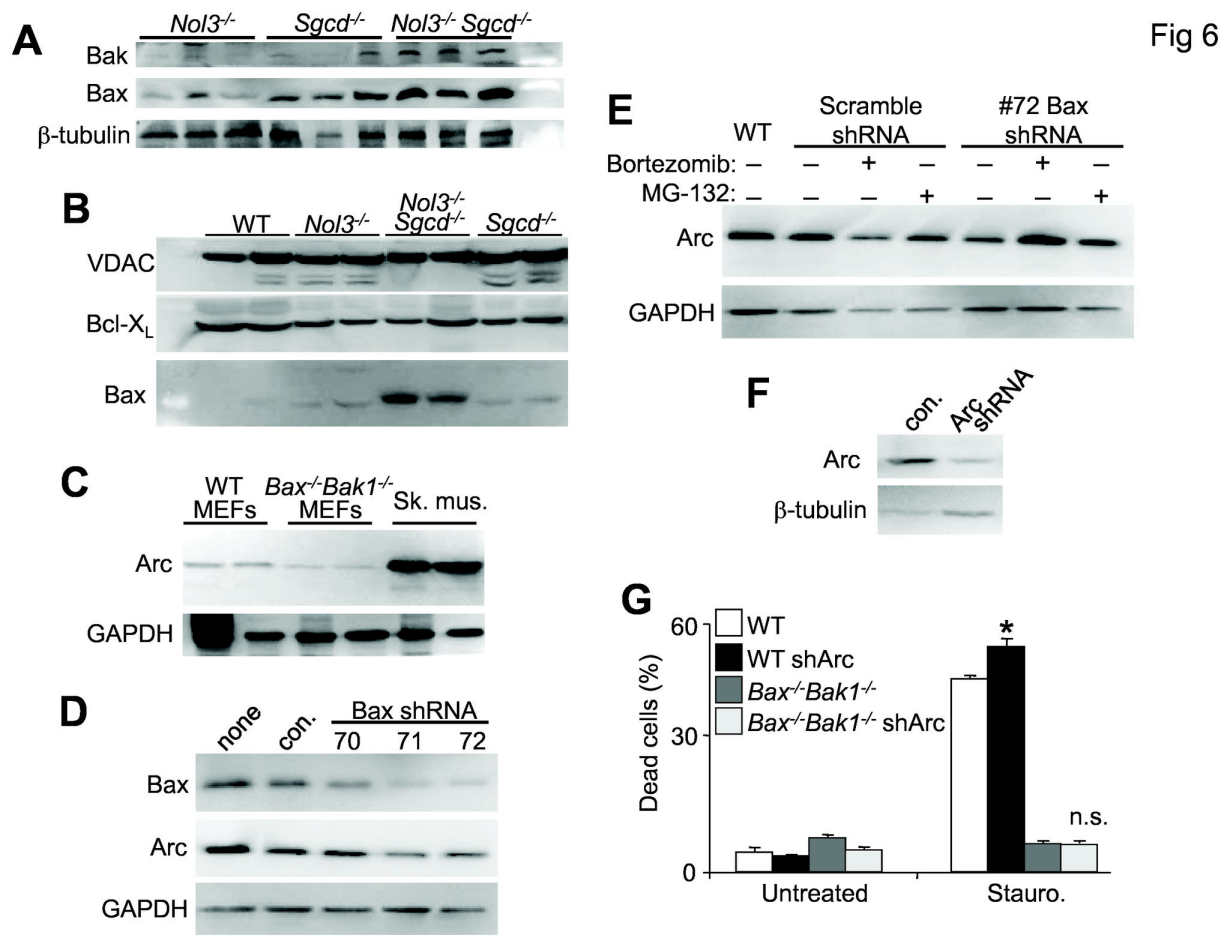
Arc deficiency increases Bax expression and cell death. A, Western blot for Bak and Bax from quadriceps lysates of Nol3^-/-^, Sgcd^-/-^, and Nol3^-/-^Sgcd^-/-^ mice. (β-tubulin serves as a loading control). B, Western blot for Bax and Bcl-X_L_ from mitochondrial protein fractions isolated from pooled hindlimb muscles of WT, Nol3^-/-^, Sgcd^-/-^, and Nol3^-/-^Sgcd^-/-^ mice. (voltage-dependent anion channel (VDAC) serves as a mitochondrial protein loading control). C, Western blotting for Arc from lysates derived from WT and Bak^-/-^Bak1^-/-^ SV40 transformed MEFs. Skeletal muscle lysates were included to show the enrichment of Arc in terminally differentiated cell types, while GAPDH serves as a protein loading control. D, Western blot for Bax and Arc in SV40 transformed MEFs infected with lentivirus expressing scrambled shRNA (con) or 3 different Bax-directed shRNAs. (GAPDH serves as a loading control). E, Western blot for Arc in SV40 transformed MEFs infected with lentivirus expressing either a scrambled shRNA or one of the Bax shRNAs and treated with proteosomal inhibitors Bortezomib or MG-132. WT MEFs are a control for normal endogenous Arc expression and GAPDH serves as a loading control. F, Western blot for Arc in SV40 transformed MEFs infected with a lentivirus expressing either a scrambled shRNA (con.) or shRNA directed against Arc. Western blots presented are quantified and statistically analyzed in Figure S3A-G. G, Quantification of dead cells by flow cytometry sorting for Annexin and PI positivity in the experimental groups shown, treated or untreated with staurosporin for 12 hours. *P<0.05 vs WT untreated. Experiment was run in triplicate.

 Given Arc’s many targets, its protective effects could be acting at several nodal points in both apoptotic and necrotic death pathways, although the zVAD-fmk results suggest that apoptosis is not a primary mechanism. Arc also directly interacts with the Bcl-2 family member Bax, which is an essential regulator of both intrinsic apoptosis [33,34] and MPTP-dependent cell death [22,23]. Western blots from 4 week-old quadriceps lysates showed no change in Bak expression (Figure S3A) but Bax expression was significantly increased (Figure S3B) in Nol3^-/-^Sgcd^-/-^ mice compared with Sgcd^-/-^ (Figure 6A). Importantly, significantly more Bax protein was detected in mitochondrial fractions isolated from muscle of Nol3^-/-^Sgcd^-/-^ mice relative to *Sgcd* ^*-/-*^ (Figure 6B and Figure S3C), suggesting that Bax activity is elevated in the double null mice. Interestingly, Arc protein abundance corresponded with the level of Bax expression as demonstrated by the decrease in Arc protein levels in *Bax-Bak1* double null MEFs, compared with WT (Figure 6C and Figure S3D). Indeed, acute knockdown of Bax in WT MEFs with three different shRNA expressing lentiviral vectors showed a dosage dependent decrease in Arc protein that matched the efficiency of Bax knockdown (Figure 6D and Figure S3E). Given that Arc is post-translationally degraded by ubiquitin-proteosomal mechanisms in response to death-stressors [35,36], we utilized two different proteasome inhibitors to block the loss of Arc protein expression in Bax knockdown MEFs (Figure 6E and Figure S3F). A 16 hour treatment with either inhibitor maintained Arc protein abundance in MEFs with nearly full knockdown of Bax (Figure 6D and 6E). To determine if Arc’s protective effects are mediated primarily through Bax, WT and *Bax* ^*-/-*^ *Bak1* ^*-/-*^ MEFs were infected with shRNAs against Arc (Figure 6F and Figure S3G) and then tested for their sensitivity to the cell death stimulus, staurosporin (Figure 6G). Arc depletion in WT MEFs caused a significant increase in cell death with staurosporin treatment (Figure 6G). By contrast *Bax* ^*-/-*^ *Bak1* ^*-/-*^ MEFs, which are resistant to cell death stimuli, were fully resistant to staurosporin even with Arc knockdown (Figure 6G). Because Arc depletion in *Bax* ^*-/-*^ *Bak1* ^*-/-*^ MEFs did not yield an additive sensitivity to staurosporin, this further supports the contention that Arc functions primarily through Bax-mediated pathways in affecting cell death.

## Discussion

 Arc antagonizes a cadre of both intrinsic and extrinsic apoptotic targets including caspase 2 and 8 [19], Fas-FADD [21], and Bax [20,21]. In dystrophic skeletal muscle Arc appears to have little influence over caspase activity as zVAD-fmk, a broad reaching caspase inhibitor, was ineffective at correcting muscle pathology in Nol3^-/-^Sgcd^-/-^ mice. This result is corroborated by data demonstrating that Arc but not zVAD-fmk blocks cytochrome *c* release in response to hypoxic stimuli [26,37]. Notably, both ischemic damaged rodent hearts and myogenic cells treated with H_2_O_2_ demonstrate a dose dependent loss of Arc that corresponded to a necrotic like phenotype characterized by the dissipation of mitochondria membrane potential and cell death [37]. In our current study myofiber death was resistant to zVAD-fmk treatment again supporting the hypothesis that Arc’s inhibitory function is upstream of the caspases in muscle and primarily dependent upon mitochondria and associated effectors. 

 Curiously, transgenic overexpression of Arc in dystrophic muscle was not protective [18]. This result is in contrast to several studies that have shown a protective effect of Arc overexpression to death stimuli in myogenic cells but also in hearts undergoing *ex vivo* Langendorff ischemia-reperfusion injury [20,26,37,38]. Perhaps the difference is the type of muscle tissue (cardiac vs. skeletal) or mode of cellular stress (hypoxic vs. Ca^2+^ overload). However, ischemia-reperfusion injury has a Ca^2+^ overload component similar to the suggested disease trigger in dystrophy [1-3], and like muscular dystrophy ischemia-reperfusion injury has dual molecular signatures of apoptosis and necrosis [39-43]. A more tenable explanation is that Arc abundance is already functionally saturated in dystrophic muscle such that more Arc expression is irrelevant in this Ca^2+^ overload context. Indeed, we observed high levels of endogenous Arc expression in skeletal muscle from *Sgcd* ^*-/-*^ mice, which differs from the significant reduction in Arc abundance that occurs during hypoxic injury or in human heart failure [24,36,37]. Global Arc deficiency can contribute to both cardiac and smooth muscle cell death after ischemic injury [24,25]. While *Nol3* null cardiac and smooth muscle showed enhanced levels of cell death in response to injury, we believe these events are primarily due to mitochondria mediated death pathways, whether that be apoptosis or MPTP-dependent necrosis [24]. 

 Apoptosis at the level of the mitochondria absolutely requires Bax and Bak [34], and recently it was suggested that Bax and Bak are also required for MPTP-dependent cell death [22,23]. Thus, Bax represents a dominant mitochondrial death effector that likely intersects with both apoptotic and necrotic cell death. Importantly, Bax directly interacts with recombinant Arc *in vitro* [20] but also with endogenous Arc in striated muscle [21]. Hence we believe that loss of Arc permits greater Bax-dependent myofiber death in the dystrophic mouse models examined here. This interpretation is also consistent with data demonstrating that Arc knockdown initiates Bax activation and apoptosis in a cardiac muscle cell line [21]. However, in skeletal muscle of *Nol3* ^*-/-*^ mice, the singular loss of Arc was not pathologic nor did it otherwise affect muscle function [24]. *Lama2* null mice were shown previously to contain abnormally high amounts of Bax-mediated skeletal muscle pathology that was significantly rescued with the genetic ablation of Bax [32]. Loss of Bax (and Bak) protein in the heart was also protective against injury providing further support for a Bax driven mitochondrial death mechanism in striated muscle [22,23,44].

Ca^2+^ overload is causal for dystrophic skeletal muscle disease, in part, by initiating MPTP formation [11]. The lowered threshold for MPTP in dystrophic muscle due to elevated Ca^2+^, in combination with greater Bax activity due to *Nol3* deficiency, sensitizes the myofibers to even greater levels of death. Whether this Bax-mediated death sensitization of skeletal muscle is due entirely to MPTP or alternative Bax functions such as outer membrane permeability and cytochrome *c* release is unknown, but inhibition of MPTP by Debio-025 partially reduced muscle pathology in Nol3^-/-^Sgcd^-/-^ mice, and *Nol3* ^*-/-*^ *Sgcd* ^*-/-*^ mitochondria were significantly swollen at baseline (Figure 5). These results are consistent with previous reports in which Debio-025 and genetic ablation of the *Ppif* gene (CypD) significantly reduced skeletal muscle disease in Sgcd^-/-^, Lama2^-/-^, and even *mdx* mice [11] underscoring the role of MPTP-dependent, programmed necrosis in dystrophic disease. Thus, we favor an overall model whereby myofiber death in adult dystrophic skeletal muscle is largely due to a regulated form of necrosis, which is consistent with histological features observed by transmission electron microscopy [9-12]. This overall conclusion does not entirely discount apoptotic pathways, which is why some apoptotic molecular markers are also elevated. Bax activity in skeletal muscle, as revealed by loss of Arc, is likely centrally involved in mediating aspects of both regulated necrosis and apoptosis. Hence, inhibitors of Bax/Bak function might offer a new therapeutic option for treating muscular dystrophy if the appropriate inhibitory agents were developed.

## Supporting Information

Figure S1 **Characterization of Arc and collagen content in quadriceps muscle from Sgcd^-/-^ and Nol3^-/-^Sgcd^-/-^ mice at 6 weeks of age.** A, Western blot for Arc from quadriceps protein lysates from wildtype (WT), Arc null (Nol3^-/-^), and *Nol3*-*Sgcd* double null (Nol3^-/-^Sgcd^-/-^) mice. (Actin serves as a loading control). B, Histological sections with picrosirus red staining from quadriceps muscle sections. Images were captured under polarized light (200x) to assess collagen content and maturity (orange color).
(EPS)

Figure S2 ***Nol3*^*-/-*^*Sgcd*^*-/-*^ mice have enhanced skeletal muscle pathology relative to *Sgcd*^*-/-*^ at 6 weeks of age.** A, Muscle weights normalized to tibial length of gastrocnemius and quadriceps, and B, serum creatine kinase (CK) levels measured from WT, *Nol3* ^*-/-*^, *Sgcd* ^*-/-*^ *, and* *Nol3* ^*-/-*^ *Sgcd* ^*-/-*^ mice. *P<0.05 vs WT; #P<0.05 vs *Sgcd* ^*-/-*^; N=8-15 per group. C, Quantification of percentage of myofibers with central nucleation from histological sections stained with H&E. D, Histological analysis of fibrotic area (blue) from sections of quadriceps stained with Masson’s trichrome and subjected to Metamorph software analysis. *P<0.05 vs WT; #P<0.05 vs *Sgcd* ^*-/-*^; N=5 per group with the identical 4 quadrants of the muscle counted per group. E, Representative Masson’s trichrome stained histological images of the data shown in D (200x) for the groups shown. F, Quantification of time to exhaustion as assessed by involuntary treadmill running. *P<0.05 vs WT; #P<0.05 vs *Sgcd* ^*-/-*^; N=4-6 per group.
(EPS)

Figure S3 **Quantification and statistical analysis of Bax and Arc expression by Western blot.** Quantification of A, Bak and B, Bax expression in quadriceps lysates from *Nol3* ^*-/-*^, *Sgcd* ^*-/-*^, and *Nol3* ^*-/-*^ *Sgcd* ^*-/-*^ mice. (β-tubulin serves as a loading control). *P<0.05 vs *Nol3* ^*-/-*^; #P<0.05 vs *Sgcd* ^*-/-*^; N=3 per group. C, Quantification of Bax and Bcl-X_L_ expression from mitochondrial protein fractions isolated from pooled hindlimb muscles of *Nol3* ^*-/-*^, *Sgcd* ^*-/-*^, and *Nol3* ^*-/-*^ *Sgcd* ^*-/-*^ mice. *P<0.05 vs *Nol3* ^*-/-*^; #P<0.05 vs *Sgcd* ^*-/-*^; †P<0.05 vs WT; N=4-6 per group. (Voltage-dependent anion channel (VDAC) serves as a mitochondrial protein loading control). D, Quantification of Arc expression in lysates derived from WT and *Bak* ^*-/-*^ *Bak1* ^*-/-*^ SV40 transformed MEFs. Skeletal muscle lysates were included to show the enrichment of Arc in terminally differentiated cell types, while GAPDH serves as a protein loading control. †P<0.05 vs WT. N=3 per group. E, Quantification of Bax and Arc expression in SV40 transformed MEFs infected with lentivirus expressing scrambled shRNA (con) or 3 different Bax-directed shRNAs. (GAPDH serves as a loading control). F, Quantification of Arc expression in SV40 transformed MEFs infected with lentivirus expressing either a scrambled shRNA or one of the Bax shRNAs and treated with proteosomal inhibitors Bortezomib or MG-132. WT MEFs are a control for normal endogenous Arc expression. (GAPDH serves as a loading control). G, Quantification of Arc expression in SV40 transformed MEFs infected with a lentivirus expressing either a scrambled shRNA (con.) or shRNA directed against Arc. (β-tubulin serves as a loading control).
(EPS)
